# Association of Viral Infection With the Development and Pathogenesis of Systemic Lupus Erythematosus

**DOI:** 10.3389/fmed.2022.849120

**Published:** 2022-02-25

**Authors:** Shigeru Iwata, Yoshiya Tanaka

**Affiliations:** First Department of Internal Medicine, University of Occupational and Environmental Health, Kitakyushu, Japan

**Keywords:** SLE, viral infection, human endogenous retroviruses, Epstein-Barr virus, B cell, immunometabolism

## Abstract

Systemic lupus erythematosus (SLE) is an autoimmune disease that causes multiple organ damage in women of childbearing age and has a relapsing-remitting course. SLE is caused by the interaction between genetic and environmental factors, however, its underlying triggers remain unknown. Among the environmental factors, the involvement of infections as a trigger for SLE, especially those of viral etiology, has been widely reported. Human endogenous retroviruses (HERVs) may put patients at a genetic predisposition to SLE, while the Epstein-Barr virus (EBV) may play a role as an environmental factor that triggers the development of SLE. It has been suggested that EBV-infected B-cells may become resistant to apoptosis, resulting in the activation, proliferation, and antibody production of autoreactive B-cells, which cause tissue damage in SLE. However, the interaction between the virus and immune cells, as well as the impact of the virus on the differentiation and dysfunction of immune cells, remain unclear. In this review, we focus on the relationship between the development and pathogenesis of SLE and viral infections, as well as the mechanism of SLE exacerbation via activation of immune cells, such as B-cells, based on the latest findings.

## Introduction

Systemic lupus erythematosus (SLE) is an autoimmune disease that causes multiple organ damage in women of childbearing age and has a relapsing-remitting course. In the pathogenesis of SLE, dysfunction of DNase I leads to impaired clearance of nucleic acids. Neutrophil extracellular traps (NETs) contain endogenous DNA released by neutrophils remaining in the body. As a result, plasmacytoid dendritic cells (pDCs) and myeloid dendritic cells (mDCs) are activated via Toll-like receptor (TLR) stimulation, leading to the production of large amounts of IFN-α. Dendritic cells induce helper T (Th) cell and B-cell differentiation as well as antibody production, either directly or through IFN-α production, resulting in progressive tissue damage by immune complex formation and deposition. Although it has been suggested that SLE is caused by the interaction of genetic and environmental factors, the underlying triggers of SLE remain unknown. Among the environmental factors, infections such as viruses, bacteria, parasites, and fungi play an important role in the development of autoimmune diseases. In particular, the involvement of viral infections in the development of SLE has been reported (1).

Viral infections have been implicated in the pathogenesis of autoimmune diseases through a variety of mechanisms, including structural or functional molecular mimicry/cross-reactivity, innate immune activation by IFN production, epigenetic factors, superantigen generation, bystander activation, regulation of apoptosis and clearance, and epitope spreading (2–14). However, the interactions between viruses and immune cells and the effects of viruses on the differentiation and dysfunction of immune cells are still unclear. In this review, we focus on the interaction of viruses with the host immune system and the mechanism of SLE exacerbation via activation of immune cells, mainly B-cells, based on the latest findings.

## Regulation Mechanism of B-Cell Differentiation and Function in SLE Pathogenesis

B-cells play an important role in autoimmune diseases through the production of antibodies and cytokines, as well as antigen presentation. Under normal conditions, self-tolerance induction mechanisms are activated during B-cell differentiation to eliminate or inactivate self-reactive B-cells that express antigen receptors (BCRs) that recognize self-antigens. Self-tolerance induction mechanisms are known to include clonal loss, receptor editing, anergy, and lack of co-stimulation and regulation by regulatory cells. One and 2 occur mainly in the central nervous system, while 3 and 4 occur mainly in the periphery (15). However, in autoimmune diseases such as SLE, self-tolerance may be disrupted by environmental factors such as viruses, in addition to genetic predisposition, with activation, proliferation, class switching, and antibody production possibly induced by external factors other than B-cells, such as dendritic cells and Th cells, as well as by endogenous dysfunction of B-cells themselves.

B-cells are activated and differentiated by co-stimulatory molecular signals, such as B-cell receptors (BCR), CD40/CD40L, Toll-like receptor (TLR) 7 and 9, as well as cytokine signals, such as IL-4, IL-21, IFN-α, and IFN-γ. As a result, BCR signals through Syk, Btk, and PLC-γ, TLR signals through MyD88, TRAF6, and NFκB, and cytokine signals through JAK-STAT are activated, and the expression of transcription factors important for differentiation, such as BACH2, BCL6, XBP1, PRDM1, and IRF4 are induced, leading to plasmablast differentiation (16–18). We have reported that activated memory B-cells [IgD-CD27-double-negative (DN) memory, class-switched memory B-cells], and plasmablasts are deeply involved in the pathogenesis of SLE (19).

Th cells include a population of cells that play a particularly important role in B-cell differentiation. Follicular helper T cells (TFHs) are widely recognized. Recently, CXCR5^−^PD1^hi^CD4^+^ peripheral helper T cells (TPH) have been reported to increase in the synovial tissue of patients with rheumatoid arthritis (20). TPHs strongly express PD-1 and induce B-cell differentiation and antibody production in an IL-21-dependent manner (21). TFH cells express BCL6 and CXCR5, while TPH cells do not express CXCR5. TPH cells do express BLIMP1 and migrate to the peripheral tissue through the expression of chemokine receptors such as CCR2, CX3CR1, and CCR5. Caielli et al. also found that CXCR5^−^CXCR3^+^PD-1^hi^CD4^+^ helper T cells, which have a different ability to induce B-cell differentiation than either TFH or TPH, are increased in both the peripheral blood of SLE patients and the tubulointerstitial region of patients with proliferative lupus nephritis (22). This population exhibited a gene expression profile that was also different from that of TFH, and induced B-cell differentiation in an IL-21, CXCL13-independent, IL-3, IL-10-dependent manner, especially with succinate. The population was found to be a distinct subset from TFH and TPH and was proposed as Th10 cells.

The effector functions of CD8^+^ T cells in SLE patients, such as granzyme B and perforin production, are impaired (23). The disease is exacerbated when perforin is deleted in lupus-prone mice (24). In a murine model of graft vs. host disease, perforin- and FasL-dependent cytotoxicity by cytotoxic T lymphocytes (CTLs) is important for the regulation of autoreactive B-cells (25, 26). Enhancing CTL cytotoxicity suppresses B-cell autoreactivity and regulates SLE disease severity (27, 28). These results suggest that CTLs may be involved in the regulation of SLE.

## Pathological Mechanism of SLE and Its Relationship to Viral Infection

Nucleic acids are involved in the pathogenesis of SLE as pathogen-associated molecular patterns (PAMPs) derived from viruses and bacteria (29–34), or damage-associated molecular patterns (DAMPs) of host origin (35–40). The immune system detects these molecules appropriately in the cytoplasm and extracellular space via pattern recognition receptors (PRRs). In the absence of infection, endogenous nucleic acids and PRRs are compartmentalized to prevent inappropriate activation of the immune system by these danger signals (41). In SLE, nucleic acids and proteins that bind to nucleic acids are the major self-antigens. The main PRRs involved in the pathogenesis of SLE are Toll-like receptor (TLR) 7 and TLR9, which recognize double-stranded RNA and DNA rich in hypomethylated CpGs, respectively (42, 43). In fact, Smith's antigen (Sm), a complex of ribonucleoprotein and non-histone nuclear RNA, as well as double-stranded DNA (dsDNA), are detected in patients with SLE (44). In SLE, PRR-mediated molecular mimicry/cross-reactivity between virus-derived nucleic acids (PAMPs) and host-derived nucleic acids (DAMPs) may lead to autoimmunity.

Overexpression of type 1 IFN-related genes, termed the “IFN signature,” has been observed in many SLE patients, and has been implicated in the etiology and pathogenesis of the disease (45). IFNα is involved in the pathogenesis and exacerbation of SLE through a variety of mechanisms, including direct and indirect effects on antigen-presenting cells, T cells, and B-cells. IFN-α is produced by plasmacytoid dendritic cells (pDCs) stimulated by viruses. IFN-α induces TLR7 expression in B-cells, and stimulation with IFN-α and TLR7 induces plasmablast differentiation (46). It has been suggested that type 1 IFN production induced by viral infection may promote these responses. Recently, the interaction between viruses and their ability to induce epigenetic modifications within the immune system has attracted attention. Epigenetic mechanisms allow the virus to create gene expression profiles that predispose the host to autoimmunity, such as altered DNA methylation in SLE susceptibility genes (e.g., IFN-related genes), histone modifications, and regulation by miRNAs (12–14).

During development of SLE, viruses produce superantigens that bind to T-cell receptor variable regions and major histocompatibility complex class II molecules. In addition, viruses promote the proliferation of autoreactive and memory T cells through a process of called “bystander activation,” which is T-cell receptor-independent and cytokine-dependent (2, 47). Viruses are involved in the survival of immune cells through the regulation of apoptosis and clearance. For example, it has been suggested that B-cells infected with EBV may become resistant to apoptosis. In addition, epitope spreading gradually leads to the production of antibodies against epitopes near the initial epitope, resulting in a diversity in antigen recognition by antibodies. As a result, self-reactive B-cells are activated, proliferate, and produce antibodies, leading to tissue damage. The impact of EBV on B-cell differentiation in the pathogenesis of SLE will be discussed in detail in Section 5.

Retroviruses, particularly human endogenous retroviruses (HERVs), have been implicated to cause genetic predisposition to SLE pathogenesis (48). In addition, viral infections with Epstein-Barr virus (EBV) may play a role as an environmental factor in triggering SLE pathogenesis (49). In particular, HERVs and EBVs involved in SLE pathogenesis are reviewed in the following sections.

## Human Endogenous Retrovirus Infection as a Trigger for SLE

Human endogenous retroviruses (HERVs) have sequences similar to those found in the germline genomes of non-virus organisms (endogenous virus-like sequences). HERVs have been stably integrated into the host mammalian genome for 40 million years (50). HERVs are recognized as self-antigens, triggering the development of autoimmune diseases. HERVs are composed of three coding regions: gag, pol, and env. It also has a long terminal repeat (LTR) containing transcriptional regulatory sequences at both ends, as well as encodes proteases and reverse transcriptases. In the human genome, only about 1.5% of gene regions have the potential to be translated into proteins, but as much as 8% of the genome is encoded by fossilized retroviruses (51). Environmental factors (infections, estrogens, drugs, chemicals, ultraviolet radiation, etc.) can epigenetically reactivate the transcription of endogenous retroviral sequences. As many as 200 endogenous retroviral sequences have been reported, some of which (HRES-1, HERV-E 4-1, HERV-K10, HERV-K18 HERVs) are related to the development of SLE through: (1) autoantibody production by molecular mimicry, (2) stimulation of intracellular sensor molecules by viral nucleic acids, and (3) epigenetic regulation of host genes (52, 53).

### Direct Stimulation of Intracellular Sensor Molecules by Viral Nucleic Acids

Binding of viral nucleic acids to intracellular sensor molecules induces the activation of a variety of pathways, all converging on a type 1 IFN response (54). Retroviruses are detected by TLRs in endosomes, which recognize dsRNA (TLR-3), ssRNA (TLR-7 and−8), and dsDNA (TLR-9) (55). In addition to TLRs in endosomes, retroviral nucleic acids are also sensitized by other PRRs in the cytoplasm (56). Although the nucleic acid sensing system is physiologically necessary to control viral infections, excessive activation may promote the progression of SLE by stimulating the production of pathogenic cytokines (57). The amount of mRNA from the HERV clone 4-1 gag region was significantly higher in SLE patients than in healthy subjects, and this amount was decreased with glucocorticoid treatment (48). However, there is no direct evidence of an association between HERV and TLRs in SLE. Further studies are warranted.

### Autoantibody Production by Molecular Mimicry

HERV proteins cross-react with self-antigens (58). For example, HRES-1 encodes the nuclear autoantigen HRES-1/p28, which is targeted by cross-reactive antiviral antibodies (59). HRES-1 also encodes HRES/Rab4, which is integrated into chromosome 1q42, the SLE susceptibility locus, and is markedly overexpressed in T cells from patients with SLE (60). Overexpression of HRES-1/Rab 4 interferes with endosomal recycling of the CD3/TCRζ chain in SLE T cells, leading to decreased expression of the receptor and lowering of the threshold for T-cell activation (61, 62). In SLE CD4+ T-cells, hypomethylation of HERV-E 4-1 terminal repeats was observed, and HERV-E 4-1 mRNA expression was higher in SLE patients than in healthy controls, which was related to disease activity (63). Anti-HERV-K envelope antibodies have been identified in patients with SLE. The envelope glycoprotein 70 (gp70) and viral nucleoprotein complexes form a retroviral gp70-anti-gp70 immune complex upon TLR7 stimulation, and induce nephropathy in mouse models (64). Thus, there are a number of reports linking HERVs to the onset and pathogenesis of SLE, albeit indirectly.

### Epigenetic Regulation of Host Immune Genes

LTRs, which are often isolated sequences, are present in large numbers in the human genome, and regulate the transcription of adjacent genes. Some LTRs contain promoter/enhancer sequences that generally have little effect on gene transcription, thus preserving their regulatory activity. However, when the epigenetic mechanisms that normally regulate them are altered, their activity is pathologically increased. This epigenetic reset can occur in autoimmunity and cancer, resulting in LTR-dependent transcription of immune genes and reactivation of HERVs (52). MER 41, an LTR belonging to the HERV family, is a STAT1 binding site located near the IFN-γ-inducible absent in melanoma 2 (*AIM2*) gene and functions as its promoter (65). Javierre et al. reported the first high-throughput and candidate sequence analyses of DNA methylation to investigate the discordance of SLE in twins. SLE patients had decreased methylation of the *AIM2* locus and increased *AIM2* expression compared to healthy twins (66). Thus, HERV may be related to the epigenetic dysregulation of *AIM2* in the pathogenesis of SLE.

## EB Virus Infection as a Trigger for SLE

EBV is latent in memory B-cells (95% of the world population). After infection by saliva, it first infects pharyngeal epithelial cells, followed by quiescent B-cells, T cells, NK cells, and neutrophils (67). Primary infection in childhood is usually asymptomatic, but it can cause infectious mononucleosis in adolescents, and is also associated with Hodgkin's lymphoma and autoimmune diseases (47, 68–72). EBV is latent in the cells of diseased individuals, but can reactivate at any time, affecting innate and acquired immunity. A relationship between pediatric SLE and EBV-DNA in peripheral blood, as well as a relationship between adult SLE and history of EBV exposure, have been reported (73). In a recent study, higher levels of antibodies against early EBV infection were detected in patients with SLE than in healthy subjects, and EBV reactivation was correlated with lymphopenia (74). Seropositivity for anti-EBV antibodies is much higher in SLE (75–77). Two recent meta-analyses have confirmed a strong association between serum anti-EBV antibody (EBNA-1 and VCA IgG) positivity and SLE development, and that prior EBV infection is a prerequisite for SLE development, at least in some ethnic groups (78, 79). On many of these grounds, EBV, in particular, is considered to be one of the potent triggers for the development of SLE (80, 81).

### Abnormalities in B-Cell Differentiation and Function Caused by EBV

It has been suggested that EBV-infected B-cells may become resistant to apoptosis. As a result, they induce the activation, proliferation, and antibody production of autoreactive B-cells, which damage tissues. EBV induces activation of naive B-cells similar to physiological signaling, such as the BCR signaling pathway via LMP (latent membrane protein) 2A (82) and CD40 stimulation via LMP1 (83). Cross-reactivity has been shown between the PPPGRRP sequence of EBNA-1 and PPPGMRPP, which may be the first epitope of the SmB antibody response, as well as between the GGSGSGPRHDGVRR sequence of EBNA-1 and TKYKQRHGWSHKD, which may be the first epitope of the anti-Ro antibody response (84). SLE-associated autoantibodies, such as anti-Sm antibodies, are commonly produced during infectious mononucleosis. Furthermore, EBV acts as a superantigen that stimulates a large number of T-cells, and exerts a number of mechanisms to induce autoimmunity.

Both lytic and latent EBV proteins elicit potent B- and T-cell responses. EBV induces high cytokine production by activated CD4 + cells, an increase in CD4 + EBV-specific cells producing IFN, and a decrease in CD8 + cell responses (85). The proliferation of EBV-infected B-cells is physiologically regulated by CD8 + cytotoxic T- cells (CTLs). However, in SLE, EBV-specific CD8 + CTLs are dysfunctional (86–88), blood EBV viral levels are chronically increased (89–91), and CTL responses by exhausted T-cells with increased PD-1 expression due to persistent EBV infection are reduced, possibly evading immune regulation (92). Sustained activation of the viral cycle increases the risk of both EBV-related malignant transformation and autoimmune disease in genetically predisposed individuals. Indeed, there is an inverse correlation between impaired function of EBV-specific CTLs and serum antibodies to soluble antigens (anti-EA/D antibodies), disease activity, and autoantibodies (86–88). Impaired EBV control by CTLs and persistent latent EBV infection are the major causes of widespread immunological changes that characterize SLE. Indeed, there are reports of 10- to 40-fold increases in EBV viral levels in peripheral blood B-cells and peripheral blood mononuclear cells from SLE patients compared to healthy controls, regardless of their receipt of immunosuppressive therapy (93–95).

### The Relevance of Immunometabolism to B-Cell Differentiation via EBV Infection

Recently, “immunometabolism” has attracted much attention. Activation and differentiation of immune cells requires massive energy production, such as ATP, and biosynthesis of bioconstituents, such as nucleic acids and lipids, by metabolic transformation. It has been reported that cellular metabolic transformation to anabolism, including aerobic glycolysis, is important for immune cell activation, mainly in mice. Metabolic pathways involving glycolysis, oxidative phosphorylation, pentose phosphate circuitry, fatty acid β-oxidation, fatty acid synthesis, and amino acid metabolism (glutaminolysis) are involved in the production of products important for cell survival and growth, and are regulated by metabolic regulators such as mTOR, AMPK, HIF1α, and c-myc (96). Studies on the involvement of intracellular metabolism in B-cell activation have been reported using mouse models as follows: enhanced PI3K-Akt-mTORC1 signaling, glycolysis, and oxidative phosphorylation induce *de novo* lipid synthesis, which is important for B-cell proliferation and growth (97). In addition, the forced expression of mTORC1 promotes plasmablast differentiation. mTORC1 is activated in B-cells of a mouse model of lupus, while rapamycin inhibits B-cell proliferation and survival (98, 99). B-cell activating factor (BAFF) and its homolog a proliferation inducing ligand (APRIL) support B-cell differentiation and plasmablast differentiation. *In vitro* studies have shown that rapamycin inhibits BAFF-mediated proliferation and survival (99). Overexpression of BAFF increased lupus-like autoantibodies and enhanced glycolysis in B-cells in a transgenic mouse model (97). However, the role of B-cell metabolism in SLE, as well as its pathogenesis, remains unknown.

EBV acts on B-cell metabolism, especially in mitochondria and inhibits apoptosis. EBV encodes proteins that target mitochondria, including BHRF1 (BamHI-H right reading frame), BZLF1 (also known as Zebra protein), BALF1 (BamHI-A left frame transcript), LMP2A, and immediate-early Zta protein. BHRF1 accumulates in the outer mitochondrial membrane (OMM) of B-cells, preventing apoptosis, promoting the survival of EBV-infected cells, and viral replication. LMP2A induces mitochondrial fission in a Drp1-dependent manner (100, 101). Immediate early Zta protein blocks mitochondrial DNA replication and promotes viral replication (102).

Mrozek-Gorska et al. confirmed that EBV also causes significant changes in the cellular content of macromolecules such as proteins and RNA, as well as increased glucose uptake, enhanced glycolysis and mitochondrial functions, and the activation of germinal center-like differentiation programs only days after infection, with transcription factors and phenotypes similar to those of plasmablasts and early plasma cells (103). Wang et al. also confirmed that EBV modifies metabolic pathways to support rapid proliferation of B-cells, confirming that EBV induces mitochondrial remodeling and carbon monoxide (1C) metabolism. EBV-encoded EBNA2 and its target MYC enhance MTHFD2 expression, a central mitochondrial 1C enzyme, and plays a critical role in B-cell survival via nucleic acid synthesis, NADPH generation, and redox maintenance (104).

### Immunometabolism in the Process of B-Cell Differentiation in SLE

**Figure 1:**
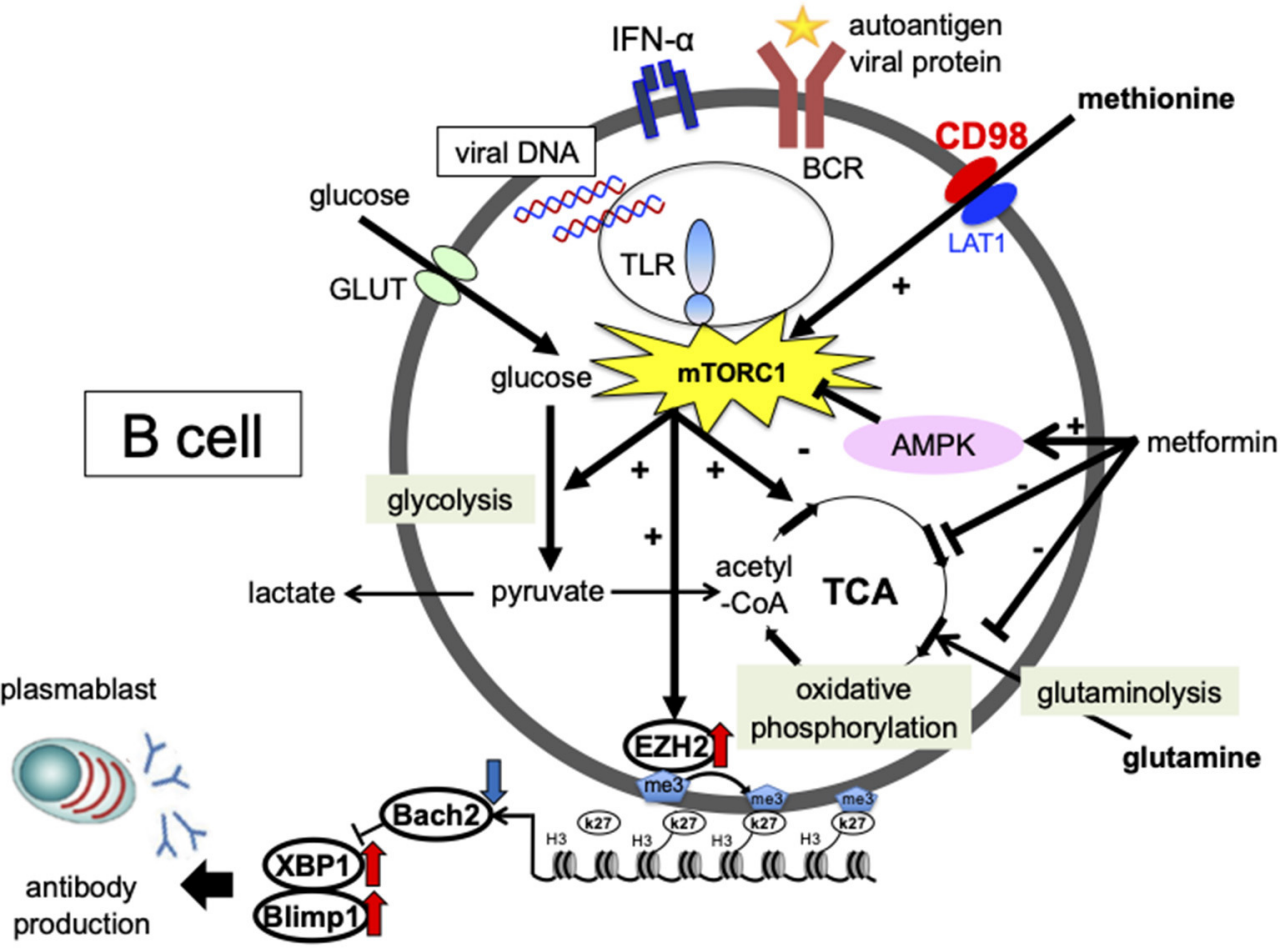
Involvement of cellular metabolism in the process of plasmablast differentiation in SLE. The glucose metabolic pathway consists of the glycolytic system, which converts glucose to pyruvate, and oxidative phosphorylation, which generates energy via the tricarboxylic acid (TCA) cycle. In the mitochondria, pyruvate is converted to acetyl-CoA, which enters the tricarboxylic acid cycle. Energy is also generated by glutamine metabolism. During anaerobic metabolism, pyruvate is converted into lactate. The essential amino acid methionine is strongly committed to plasmablast differentiation. In the presence of methionine, mTORC1 activation induces the expression of the methyltransferase enhancer zeste homolog 2 (EZH2). EZH2 induces H3K27me3 at BTB and CNC homolog 2 (BACH2) loci and suppresses BACH2 expression, leading to the induction of B lymphocyte-induced maturation protein-1 (BLIMP1) and X-box binding protein 1 (XBP1) expression and plasmablast differentiation. Metformin inhibited CpG- and IFN-α-induced glutamine uptake, mitochondrial function, and suppressed plasmablast differentiation. Enhanced cellular metabolism mediated by amino acids, such as methionine and glutamine, is important for plasmablast differentiation, which may be a potential therapeutic target for SLE.

EBV-induced expression of the latent protein LMP2A activates the PI3K/Akt/mTORC1 pathway (105, 106). However, the mechanism of activation and differentiation via mTOR in B-cells, and its involvement in the pathogenesis of SLE, remains unclear. We investigated the effects of IFN-α and a nucleic acid on the intracellular metabolism and differentiation of B-cells from SLE patients. p-mTOR expression levels were increased in B-cells from SLE patients compared to healthy controls and was correlated with the percentage of plasmablasts and disease activity (107). Activation of mTORC1 is regulated by various environmental signals, including amino acid levels. We found that methionine, an essential amino acid, is important for CpG- and IFN-α-induced plasmablast differentiation. In the presence of methionine, activation of mTORC1 induces the methyltransferase EZH2, which represses BACH2 expression via induction of H3K27me3 in the gene expression region of BACH2, consequently promoting BLIMP1 and XBP1 expression and plasmablast differentiation (Figure 1). B-cells from SLE patients overexpressed EZH2, which correlated with disease activity and autoantibody production (108) (Figure 1), and the expression level of DiOc6, which indicates hyperpolarization of the mitochondrial membrane, was higher in B-cells from SLE patients than in healthy subjects. *In vitro* stimulation of CD19 + cells with CpG and IFNα-induced plasmablast differentiation led to increased glycolysis, oxidative phosphorylation, and DiOc6 expression. The glutaminase inhibitor BPTES, which is a limited inhibitor of glutamine degradation, decreased DiOc6 expression, oxidative phosphorylation, ROS production, and ATP production, as well as suppressed plasmablast differentiation and antibody production. Metformin inhibited CpG- and IFNα-induced glutamine uptake, as well as suppressed plasmablast differentiation and antibody production (Figure 1) (109). In SLE patients, B-cells are activated and differentiated by viral and autoantigen-derived nucleic acids and IFN-α stimulation through the uptake of amino acids such as methionine and glutamine, which are related to the pathogenesis of SLE.

## Discussion

Viral infections are important environmental factors in the pathogenesis of SLE, and can trigger disease onset and relapse, as well as play an important role in altering clinical phenotypes. A wide range of viral-mediated immunomodulatory mechanisms have become increasingly evident. These mechanisms can profoundly alter the host immune system and interact to influence B-cell differentiation in SLE pathogenesis, such as the “immortalization” of autoreactive B-cells by EBV. Studies on the epigenetic regulation of IFN-responsive genes by reactivated HERVs have provided important insights into the missing link between environmental factors and genetic predisposition. In addition to hydroxychloroquine (HCQ), mycophenolate mofetil, anti-BAFF antibody belimumab, the anti-IFNAR antibody aniflorumab has been used for the treatment of SLE. Regarding the effect of HCQ on viral infections, HCQ has been shown to inhibit viral binding to receptors and entry into cells by inhibiting glycosylation and increasing pH during phagocytosis. However, in recent years, HCQ has not been recommended as a therapeutic agent for COVID-19 due to the lack of efficacy demonstrated for its effect on COVID-19 and many reports of concerns about side effects. In addition, aniflorumab is expected to be effective against SLE. However, there is also concern about the increased risk of viral infections because it suppresses the antiviral effects of type 1 IFN. Thus, a deeper understanding of the interaction between viral proteins and nucleic acids and the host's immune system is essential for the best use of therapeutic agents, such as TLR inhibitors, aniflorumab, and belimumab, as well as in considering the new concept of immunometabolism.

## Author Contributions

SI wrote the manuscript. YT reviewed and edited the manuscript. Both authors contributed to the article and approved the submitted version.

## Funding

This work was supported in part by JSPS KAKENHI grant number #JP16K09928 and the University of Occupational and Environmental Health, Japan, through the UOEH Grant for Advanced Research (#H29-903 and #H30-905).

## Conflict of Interest

YT received research grants from Mitsubishi-Tanabe, Takeda, Daiichi-Sankyo, Chugai, Bristol-Myers, MSD, Astellas, Abbvie, and Eisai. The remaining author declare that the research was conducted in the absence of any commercial or financial relationships that could be construed as a potential conflict of interest.

## Publisher's Note

All claims expressed in this article are solely those of the authors and do not necessarily represent those of their affiliated organizations, or those of the publisher, the editors and the reviewers. Any product that may be evaluated in this article, or claim that may be made by its manufacturer, is not guaranteed or endorsed by the publisher.

## Glossary

SLE; Systemic lupus erythematosus

HERVs; Human Endogenous Retroviruses

EBV; Epstein-Barr virus

pDCs; Plasmacytoid dendritic cells

mDCs; Myeloid dendritic cells

TLR; Toll-like receptor

Th; T helper

IFN; interferon

BCRs; B cell receptors

IL; interleukin

TFH; Follicular helper T cells

TPH; Peripheral helper T cells

CTLs; Cytotoxic T cells

PAMPs; Pathogen-associated molecular pattern molecules

DAMPs; damage-associated molecular patterns

PRRs; pattern-recognition receptors

dsDNA; double-stranded DNA

LTR; long terminal repeat

AIM2; absent in melanoma 2

LMP; latent membrane protein

BAFF; B Cell Activating Factor

OMM; outer mitochondrial membrane

HCQ; Hydroxychloroquine

IFNAR; interferon-α/β receptor
